# The relationship between physical literacy scores and adherence to Canadian physical activity and sedentary behaviour guidelines

**DOI:** 10.1186/s12889-018-5897-4

**Published:** 2018-10-02

**Authors:** Kevin Belanger, Joel D. Barnes, Patricia E. Longmuir, Kristal D. Anderson, Brenda Bruner, Jennifer L. Copeland, Melanie J. Gregg, Nathan Hall, Angela M. Kolen, Kirstin N. Lane, Barbi Law, Dany J. MacDonald, Luc J. Martin, Travis J. Saunders, Dwayne Sheehan, Michelle Stone, Sarah J. Woodruff, Mark S. Tremblay

**Affiliations:** 1 Healthy Active Living and Obesity (HALO) Research Group, Children’s Hospital of Eastern Ontario Research Institute, 401 Smyth Road, Ottawa, ON K1H 8L1 Canada; 2 Centre for Sport and Exercise Education, Camosun College, Victoria, BC V9E 2C1 Canada; 3 School of Physical and Health Education, Nipissing University, North Bay, ON P1B 8L7 Canada; 4 Department of Kinesiology and Physical Education, University of Lethbridge, Lethbridge, AB T1K 3M4 Canada; 5 Department of Kinesiology and Applied Health, University of Winnipeg, Winnipeg, MB R3B 2E9 Canada; 6 Department of Human Kinetics, St. Francis Xavier University, Antigonish, NS B2G 0W5 Canada; 7 Department of Applied Human Sciences, University of Prince Edward Island, Charlottetown, PEI C1A 4P3 Canada; 8 School of Kinesiology and Health Studies, Queen’s University, Kingston, ON K7L 3N6 Canada; 9 Faculty of Health, Community and Education, Mount Royal University, Calgary, AB T3E 6K6 Canada; 10 School of Health and Human Performance, Dalhousie University, Halifax, NS B3H 4R2 Canada; 11 Department of Kinesiology, University of Windsor, Windsor, ON N9B 3P4 Canada

**Keywords:** Physical literacy, Physical activity, Sedentary behaviour, Children

## Abstract

**Background:**

Physical literacy is an emerging construct in children’s health promotion, and may impact their lifelong physical activity habits. However, recent data reveal that only a small portion of Canadian children are regularly physically active and/or meet sedentary behaviour guidelines. To our knowledge, no study has investigated the association between physical literacy and movement behaviour guidelines. Therefore, the purpose of this study was to examine the relationship between physical literacy scores in Canadian children who meet or do not meet physical activity and sedentary behaviour guidelines.

**Methods:**

Children (*n* = 2956; 56.6% girls) aged 8–12 years from 10 Canadian cities had their physical literacy levels measured using the Canadian Assessment of Physical Literacy, which consists of four domains (Physical Competence; Daily Behaviour; Knowledge and Understanding; and Motivation and Confidence) that are aggregated to provide a composite physical literacy score. Physical activity levels were measured by pedometers, and sedentary behaviour was assessed through self-report questionnaire. Analyses were conducted separately for each guideline, comparing participants meeting versus those not meeting the guidelines. Comparisons were performed using MANOVA and logistic regression to control for age, gender, and seasonality.

**Results:**

Participants meeting physical activity guidelines or sedentary behaviour guidelines had higher physical literacy domain scores for Physical Competence and for Motivation and Confidence compared to those not meeting either guideline (both *p* < 0.0001). Participants had increased odds of meeting physical activity guidelines and sedentary behaviour guidelines if they met the minimum recommended level of the Physical Competence and Motivation and Confidence domains. Significant age (OR 0.9; 95% CI: 0.8, 0.9), gender (OR 0.4; 95% CI: 0.3, 0.5) and seasonality effects (OR 1.6; 95% CI: 1.2, 2.2 spring and OR 1.7; 95% CI: 1.2, 2.5 summer, reference winter) were seen for physical activity guidelines, and age (OR 0.8; 95% CI: 0.7, 0.8) and gender effects (OR 1.7; 95% CI: 1.4, 2.0) for sedentary behaviour guidelines. Knowledge and Understanding of physical activity principles was not related to guideline adherence in either model.

**Conclusions:**

These cross-sectional findings demonstrate important associations between physical literacy and guideline adherence for physical activity and sedentary behaviour. Future research should explore the causality of these associations.

## Background

Regular participation in physical activity is recommended for children and adolescents in order for them to achieve and maintain a healthy lifestyle. For North American children and youth, it is recommended that they engage in at least 60 min of moderate - to vigorous - intensity physical activity each day [1, 2]. Simultaneously, health practitioners recommend that children and youth reduce the time spent being sedentary by limiting screen time to no more than 2 hours a day in Canada [1], and less than one to 2 hours a day in the United States [3].

There are many factors that contribute to the achievement of a healthy, active lifestyle and meeting physical activity guidelines (PAG) and sedentary behaviour guidelines (SBG); however, one area that has recently gained attention in the field of healthy active living is physical literacy (PL). The International Physical Literacy Association defines PL as “the motivation, confidence, physical competence, knowledge and understanding to value and take responsibility for engagement in physical activities for life” [4]. Children who are physically literate are capable of moving with confidence and competence in a wide variety of physical activities in multiple environments (e.g., land, snow, water, ice) [5]. Possessing an elevated level of PL may enable children to engage in habitual physical activity and reduce their sedentary time. Conversely, children with low levels of PL may engage in insufficient physical activity to receive the health benefits associated with meeting PAG [6] and may potentially experience the deleterious effects of excessive sedentary behaviour [3].

PL is considered a dynamic concept, and has been regarded as a “lifelong journey” [7]. However, the assessment of PL levels in children may be a key period for both research and intervention, as this stage in a child’s life is a critical period for the development of important physical activity correlates (i.e., gross-motor skills, fine-motor skills, coordination, preferences, and confidence). The Canadian Assessment of Physical Literacy (CAPL) [8] was developed to comprehensively and accurately measure PL, while adhering to the internationally accepted definition of the concept [4]. The CAPL’s development was guided and finalized by a three-round Delphi expert review process to ensure that the model, evaluation metrics, and measurement procedures effectively and reliably assess PL [8–19].

Though physical activity and sedentary behaviour in children are well-studied areas in the field of health promotion, there has not been thorough exploration of possible linkages between the four domains of PL and children who meet or do not meet PAG and SBG. Therefore, the purpose of this study was to examine associations between PL domain scores among children who meet or do not meet Canadian PAG or SBG. It was hypothesized that children meeting PAG and SBG would demonstrate higher PL domain scores compared to children not meeting the guidelines.

## Methods

### Study design

The Royal Bank of Canada Learn to Play – Canadian Assessment of Physical Literacy (RBC Learn to Play – CAPL) study is a cross-sectional, national surveillance study designed to evaluate the PL of Canadian children. The overarching RBC Learn to Play – CAPL study collected data in 11 cities across seven Canadian provinces using convenience sampling methods. The overall study was initially approved by the Children’s Hospital of Eastern Ontario Research Ethics Board (coordinating centre), and was subsequently approved by each site’s institutional Research Ethics Board and local school boards. All children provided verbal assent to participate in the study, and written informed consent was provided by a parent/guardian.

### Participants and setting

Canadian children aged 8–12 years from 10 cities (Victoria, British Columbia; Lethbridge, Alberta; Calgary, Alberta; Winnipeg, Manitoba; North Bay, Ontario; Windsor, Ontario; Ottawa, Ontario; Halifax, Nova Scotia; Antigonish, Nova Scotia; and Charlottetown, Prince Edward Island) from the RBC Learn to Play – CAPL study were included in this analysis. (Trois-Rivières, Québec, the 11th city involved in the RBC Learn to Play – CAPL study, was not eligible for this analysis because none of their participants had ≥6 days of valid pedometer data.) Study Site Investigators were recruited through professional networks of the Principal Investigator (MST), with a focus on selecting individuals in key geographic regions of Canada. The primary recruitment locations for this study were Canadian primary schools, with efforts to capture data from participants of varying socio-economic status and different residential living locations (i.e., urban, rural, suburban). After receiving approval from local school boards, Site Investigators contacted school principals via email using a standardized recruitment letter developed by the coordinating centre (Ottawa, Ontario). Secondary recruitment locations included summer camps, community centres, and sport clubs located in each respective city/area.

### Measurements

Data collection was performed year-round beginning in the spring of 2014 up until winter 2017; seven sites began in 2014 and the remaining three sites began in 2015. Personnel from all sites participating in the RBC Learn to Play - CAPL study were trained on how to administer the CAPL by research staff from the coordinating centre (Ottawa, Ontario). Specifically, this involved all Site Investigators attending a two-day training workshop at the coordinating centre, where the overall Project Coordinator explained the development of the CAPL and how each measure was to be administered and scored, and where each Site Investigator was trained by performing mock data collection on volunteers. Data were collected at each site by trained Site Investigators, research assistants and/or post-secondary students following standardized procedures for each of the CAPL’s four domains (Physical Competence, Daily Behaviour, Knowledge and Understanding, Motivation and Confidence), which were then aggregated to provide a composite PL score.

The composite PL score is out of 100, and scores are assigned to each of four categories for interpretation: Beginning (children have not yet achieved an acceptable level of PL); Progressing (improved PL score but have not yet achieved an acceptable level of PL); Achieving (obtained a score reflective of sufficient PL); and Excelling (demonstrated a high level of PL) [8]. The minimum recommended level of PL defined by the CAPL’s scoring system is the ‘Achieving’ category. The Daily Behaviour domain was not included in analyses that categorized participants who met PAG/SBG or predicted PAG/SBG adherence, as a sizable portion of this domain’s scoring structure is influenced by meeting both guidelines. A summary of the CAPL protocols and measures is provided in Table 1; detailed explanations of each protocol can be found in the literature [8] and in the CAPL Manual online (https://www.capl-ecsfp.ca/capl-manual/).

**Table 1:** Summary table of CAPL protocols

Domain	Protocol [ref]	Description	Units	Scoring
Physical Competence	Plank [10]	Hold prone plank position for long as possible, to measure torso muscular endurance and strength	sec	Nearest 0.1 s
	PACER 15 m/20 m [11]	Run as many laps as possible, to measure aerobic fitness	laps	Total laps recorded
	Handgrip strength [12]	Squeeze dynamometer with each hand twice, to provide an indication of upper-body strength	kg	Best score from each hand combined
	Sit and reach [12]	Seated with shoes removed, legs straight and bent forward with one hand over the other, to measure hamstring and trunk flexibility	cm	Highest value from two trials recorded
	CAMSA [13]	Measures the quality and speed of fundamental movement skills and complex movement skills	sec and criteria	Highest value from two trials recorded
	Height [8]	Footwear removed and height measured with a portable dynamometer	cm	Units recorded
	Weight [8]	Footwear removed and weight measured with digital scale	kg	Units recorded
	Waist circumference [8]	Measured at the mid-axillary line just above the iliac crest	cm	Units recorded
Daily Behaviour	Pedometer [14, 15]	Worn for 7 days for a minimum of 10 h/day, to determine physical activity guideline adherence	steps/day	Days meeting guidelines recorded
	Screen time [16]	Self-reported, to measure weekday and weekend screen time for sedentary behaviour	hrs/day	Days meeting guidelines recorded
Knowledge and Understanding	PA questionnaire [17]	Comprehension of healthy living principles based on PHE curricula	items	Questionnaire items scored
Motivation and Confidence	Questionnaire [18, 19]	Level of adequacy and predilection in relation to PA; benefits and barriers to PA also measured.	items	Questionnaire items scored

*CAMSA* Canadian Agility and Movement Skill Assessment; *CAPL* Canadian Assessment of Physical Literacy; *cm* Centimetre; *hrs* Hours; *kg* Kilogram; *PA* Physical activity; *PACER* Progressive Aerobic Cardiovascular Endurance Run; *PHE* Physical health and education; *Ref* Reference; *sec* Seconds

### Data treatment

Only participants with at least 6 valid days of pedometer data (step counts between 1000 and 30,000/day; wear time of ≥10 h/day; completed daily pedometer log sheet) were eligible for analysis [14]. Participants were classified as either meeting PAG (≥12,000 steps ≥6 days/week) [20] or not meeting PAG. For SBG, participants were classified as either meeting the guidelines (≤2 h screen time/day) [21] or not meeting the guidelines. Participants self-reported their screen time as a proxy for sedentary behaviour, with questions selected from the United States’ Youth Risk Behavior Surveillance System [16]. Participants reported (1) how many hours they typically watched TV, and (2) how many hours they played video games and/or used a computer during leisure time; these two questions were asked separately for both weekdays and weekend days. Available responses were: 0 = I did not watch TV, 0.5 = ≤1 h of TV, 1 = 1 h, 2 = 2 h, 3 = 3 h, 4 = 4 h, 5 = 5 or more hours. A weighted mean score was calculated based on the following formula: [(hours of TV weekdays × 5) + (hours of TV weekend × 2) + (hours of video games and/or computer use weekdays × 5) + (hours of video games and/or computer use weekend × 2)] /7. Participants with a weighted score ≤ 2.0 would be considered meeting SBG.

### Statistical analysis

Descriptive statistics and PL domain scores were calculated as means and standard deviations for participants, stratified by those meeting and not meeting PAG and SBG (comparisons for PAG and SBG were kept distinct from the other). MANOVAs were used to compare differences in descriptive statistics. Cohen’s *d* was calculated to determine effect sizes between descriptive comparisons. Logistic regressions were used to test for differences in PL domain scores between those meeting and not meeting PAG and SBG, while controlling for age, gender, and seasonality. Odds ratios were calculated for participants from logistic regressions, and McFadden R^2^ values were generated for each guideline’s model. Statistical analyses were conducted using R version 3.4.1 (The R Foundation for Statistical Computing, Vienna, Austria). Several packages were loaded into R for use with the analyses, including “psych”, “effsize”, “lubridate”, “rms”, and “pscl”. Significance was set at *p* < 0.05.

## Results

The descriptive characteristics of the study sample are provided in Tables 2 and 3. Participants were 2956 Canadian children (56.6% girls). Boys had significantly higher PL domain scores and overall PL scores than girls (all *p* < 0.001) except for the Knowledge and Understanding domain, where girls scored higher than boys (*p* = 0.0001). However, effect sizes for most of these comparisons were considered negligible (all < 0.2), except for Daily Behaviour (Cohen’s *d* = 0.22) and overall PL (Cohen’s *d* = 0.20), for which the effect sizes were considered small.

**Table 2:** Descriptive characteristics of participants stratified by data collection site (*n* = 2956)

Study site	Sample size (n)	Age (yr)	Gender
Alberta			
Calgary	543	11.1 (1.1)	236 boys (43%), 307 girls (57%)
Lethbridge	206	10.9 (1.0)	91 boys (44%), 115 girls (56%)
British Columbia			
Victoria	99	10.2 (0.9)	48 boys (48%), 51 girls (52%)
Ontario			
North Bay	385	10.2 (1.2)	160 boys (42%), 225 girls (58%)
Ottawa	354	11.0 (1.1)	164 boys (46%), 190 girls (54%)
Windsor	141	10.2 (1.2)	62 boys (44%), 79 girls (56%)
Manitoba			
Winnipeg	359	11.1 (1.0)	155 boys (43%), 204 girls (57%)
Nova Scotia			
Antigonish	469	10.3 (1.2)	206 boys (44%), 263 girls (56%)
Halifax	293	9.9 (1.2)	122 boys (42%), 171 girls (58%)
Prince Edward Island			
Charlottetown	107	10.6 (1.1)	39 boys (36%), 68 girls (64%)

Data are shown as mean (SD) where appropriate. Sites are listed by Canadian province, followed by specific study site(s)

**Table 3:** Descriptive characteristics of participants stratified by gender (*n* = 2956)

	Boys (*n* = 1283)	Girls (*n* = 1673)	Cohen’s *d*	*p*
Age, yr	10.6 (1.2)	10.6 (1.2)	0.01	0.77
Physical Competence (/32)	20.2 (4.4)	19.5 (4.1)	0.17	< 0.0001
Daily Behaviour (/32)	19.7 (6.1)	18.4 (5.8)	0.22	< 0.0001
Motivation and Confidence (/18)	12.9 (2.7)	12.4 (2.5)	0.18	< 0.0001
Knowledge and Understanding (/18)	11.9 (2.8)	12.3 (2.6)	−0.14	0.0001
Physical Literacy (/100)	64.6 (11.0)	62.5 (10.0)	0.20	< 0.0001

Data are shown as mean (SD). *p* value for differences between genders (MANOVA test)

Effect sizes were considered negligible if < 0.2, small if between 0.2–0.5, moderate if between 0.5–0.8, and important if > 0.8

The proportion of participants meeting PAG and SBG is presented in Table 4. MANOVA revealed that only 20% of participants met PAG (*n* = 577), with a significantly higher proportion of boys (27%) meeting PAG than girls (14%; *p* < 0.0001). Those meeting PAG displayed significantly higher Physical Competence and Motivation and Confidence domain scores than those not meeting the guidelines (both *p* < 0.0001). Only small effect sizes were seen for Physical Competence (Cohen’s *d* = 0.44) and Motivation and Confidence (Cohen’s *d* = 0.39). Regarding SBG, 57% of participants (*n* = 1633) reported meeting the guidelines, with a significantly higher proportion of girls (62%) meeting the SBG compared to boys (50%; *p* < 0.0001). Similar to PAG, those meeting SBG had significantly higher Physical Competence and Motivation and Confidence domain scores compared to those not meeting the guidelines (both *p* < 0.0001). Only small effect sizes were revealed for the Physical Competence (Cohen’s *d* = 0.21) and Motivation and Confidence (Cohen’s *d* = 0.42) domains. Knowledge and Understanding domain scores were not associated with guideline adherence.

**Table 4:** Descriptive characteristics of participants stratified by adherence to Canadian physical activity guidelines (PAG) (*n* = 2956) and sedentary behaviour guidelines (SBG) (*n* = 2879)

	Meeting PAG (*n* = 577)	Not meeting PAG (*n* = 2379)	Cohen’s *d*	*p*
Age, yr	10.5 (1.2)	10.6 (1.2)	−0.13	0.01
Physical Competence (/32)	21.3 (4.2)	19.4 (4.2)	0.44	< 0.0001
Motivation and Confidence (/18)	13.3 (2.3)	12.4 (2.6)	0.39	< 0.0001
Knowledge and Understanding (/18)	12.1 (2.7)	12.1 (2.7)	0.01	0.51
	Meeting SBG (*n* = 1633)	Not meeting SBG (*n* = 1246)	Cohen’s *d*	*p*
Age, yr	10.5 (1.2)	10.8 (1.1)	−0.28	< 0.0001
Physical Competence (/32)	20.2 (4.3)	19.3 (4.2)	0.21	< 0.0001
Motivation and Confidence (/18)	13.1 (2.4)	12.0 (2.7)	0.42	< 0.0001
Knowledge and Understanding (/18)	12.1 (2.6)	12.2 (2.8)	−0.04	0.42

Data are shown as mean (SD)

*p* value for differences between those meeting physical activity guidelines and sedentary behaviour guidelines versus those not meeting the guidelines (MANOVA test)

Chi-squared test revealed more boys (27%) met PAG than girls (14%) (*p* < 0.0001)

Chi-squared test revealed more girls (62%) met SBG than boys (50%) (*p* < 0.0001)

Effect sizes were considered negligible if < 0.2, small if between 0.2–0.5, moderate if between 0.5–0.8, and important if > 0.8

Physical activity guideline adherence was defined as attaining ≥12,000 steps, measured by pedometer, on ≥6 days/week [17]. Sedentary behaviour guideline adherence was defined as ≤2 h screen time/day on weekdays and weekends [10, 11]

A Pearson correlation coefficient of − 0.10 (*p* < 0.0001) was calculated between physical activity step counts and hours of screen time, demonstrating that these variables are not strongly correlated

Data from the logistic regression are presented in Table 5, with odds ratios derived for each separate guideline analysis. Participants had greater odds of meeting PAG if they demonstrated the minimum recommended level of Physical Competence (OR 2.1; 95% CI: 1.7, 2.5) and Motivation and Confidence (OR 1.2; 95% CI: 1.0, 1.5). No significant findings were identified for the Knowledge and Understanding domain. Girls were at decreased odds (OR 0.4; 95% CI: 0.3, 0.5) of meeting PAG compared to boys. Age was also a significant predictor of PAG adherence; with each one-year increment increase in age, participants had slightly lower odds (OR 0.9; 95% CI: 0.8, 0.9) of meeting PAG. Significant effects for seasonality were observed, demonstrating that participants who were tested in the spring (OR 1.6; 95% CI: 1.2, 2.2) and summer months (OR 1.7; 95% CI: 1.2, 2.5) were at increased odds of meeting PAG compared to participants tested in the winter months.

**Table 5:** Logistic regression analyses for associations between physical literacy domains and physical activity guideline (PAG) adherence and sedentary behaviour guideline (SBG) adherence

	B	SE	Z ratio	*p*	OR (95% CI)
PAG model					
Intercept	−0.206	0.448	−0.46	0.6457	
Physical Competence	0.726	0.104	6.99	< 0.0001	2.1 (1.7, 2.5)
Motivation and Confidence	0.218	0.105	2.07	0.0385	1.2 (1.0, 1.5)
Knowledge and Understanding	−0.104	0.105	−0.99	0.3234	0.9 (0.7, 1.1)
Age	−0.146	0.044	−3.29	0.0010	0.9 (0.8, 0.9)
Gender	−0.898	0.102	−8.83	< 0.0001	0.4 (0.3, 0.5)
Season when tested					
Spring	0.492	0.146	3.38	0.0007	1.6 (1.2, 2.2)
Summer	0.538	0.196	2.74	0.0061	1.7 (1.2, 2.5)
Fall	0.262	0.153	1.72	0.0864	1.3 (1.0, 1.8)
SBG model					
Intercept	2.053	0.369	5.57	< 0.0001	
Physical Competence	0.389	0.086	4.51	< 0.0001	1.5 (1.2, 1.7)
Motivation and Confidence	0.729	0.089	8.22	< 0.0001	2.1 (1.7, 2.5)
Knowledge and Understanding	−0.093	0.084	−1.11	0.2680	0.9 (0.8, 1.1)
Age	−0.239	0.036	−6.58	< 0.0001	0.8 (0.7, 0.8)
Gender	0.509	0.081	6.25	< 0.0001	1.7 (1.4, 2.0)
Season when tested					
Spring	−0.038	0.111	− 0.34	0.7330	1.0 (0.8, 1.2)
Summer	0.164	0.157	1.04	0.2970	1.2 (0.9, 1.6)
Fall	0.031	0.118	0.26	0.7940	1.0 (0.8, 1.3)

Reference categories for the physical literacy domains (physical competence, motivation and confidence, knowledge and understanding), gender and season when tested are for those not meeting the recommended levels, boys, and winter, respectively

McFadden R^2^ values for the PAG and SBG models are 0.15 and 0.12, respectively

Domain scores (physical competence, motivation and confidence, knowledge and understanding) were analyzed as meeting the minimum recommended level as defined by the CAPL

Similar to PAG, participants had higher odds of meeting SBG if they achieved the minimum recommended level of Physical Competence (OR 1.5; 95% CI: 1.2, 1.7) and Motivation and Confidence (OR 2.1; 95% CI: 1.7, 2.5). No significant findings were observed for the Knowledge and Understanding domain. Girls were at increased odds (OR 1.7; 95% CI: 1.4, 2.0) of meeting SBG compared to boys. Age was also a significant predictor of SBG adherence; with each one-year increment increase in age, participants had slightly lower odds (OR 0.8; 95% CI: 0.7, 0.8) of meeting SBG. The PAG model explained slightly more variance than the SBG model (PAG: McFadden *R*^*2*^ = 0.15; SBG: McFadden *R*^*2*^ = 0.12).

## Discussion

The purpose of this study was to determine if there were associations between PL scores and PAG and SBG adherence. Logistic regression, performed separately for each guideline, revealed that children were at increased odds of meeting PAG and SBG if they met the minimum recommended level of Physical Competence and Motivation and Confidence. Specifically, the Physical Competence domain was shown to be the strongest predictor in the PAG model, while Motivation and Confidence appeared as the stronger predictor in the SBG model. No significant findings were observed for the Knowledge and Understanding domain.

It is difficult to draw direct linkages to previous findings in the literature since this is the first study, to our knowledge, that collectively compares the domains of PL to children’s PAG and SBG adherence. However, our data are supported by previous research that examined aspects of PL in isolation. For example, Morrow et al. showed that children not meeting PAG were less likely to achieve healthy physical fitness levels [22]. We found Physical Competence domain scores were lower among children who did not meet Canadian PAG. Similarly, Larouche et al. demonstrated that daily physical activity behaviour measured by pedometer was significantly correlated with CAPL health-related fitness measures (aerobic power, handgrip strength, plank) and motor skills as determined by the Canadian Agility and Movement Skill Assessment (CAMSA) [23]. Children in our study were more likely to meet PAG if they met the minimum recommended level of Physical Competence. Previous research also illustrated that fundamental movement skills (FMS) play a critical role in physical activity engagement in childhood and adolescence [24, 25], which is consistent with the CAPL incorporating a measure of FMS (i.e., CAMSA) into the Physical Competence domain. Our findings showed that the largest effect size observed when comparing PL domain scores between those meeting and not meeting PAG was in the Physical Competence domain (Cohen’s *d* = 0.44), which suggests the importance of having adequate physical abilities to meet PAG. However, because our data was cross-sectional, we cannot infer causality between a child’s physical competence and PAG adherence.

Regarding SBG, recent research by Edelson et al. showed that screen time, specifically TV time, was negatively associated with functional strength measures in a national sample of American children [21]. Additionally, research looking at sedentary time and motor coordination in isolation found that boys and girls who spent less than 76.5 and 77.3%, respectively, of their waking hours being sedentary had increased odds of achieving normal or good motor coordination scores [26]. This is consistent with findings from our Canadian study, as children who did not meet SBG had significantly lower levels of Physical Competence; yet the effect sizes for SBG adherence were half of what was observed for PAG adherence (Cohen’s *d* = 0.21). Nonetheless, reducing time spent sedentary and replacing it with time spent in physical activities may be an advantageous approach to enhance a child’s PL.

Results from this study demonstrated that children meeting PAG and SBG had significantly higher scores in the Motivation and Confidence domain compared to children not meeting the guidelines. Additionally, children were at increased odds of meeting PAG and SBG when they met the minimum recommended level of Motivation and Confidence. A systematic review by Owen et al. revealed that increased levels of motivation were related to greater physical activity [27]. However, research has suggested that the motivation to be active is independent of the motivation to be sedentary [28]. Further identifying the interplay between motivation, physical literacy, and sedentary behaviour may be an area of research that warrants exploration, as the Motivation and Confidence domain was the strongest predictor in the SBG model.

Although these findings align with the stated hypothesis, no significant differences or predictor statuses were observed in any of our analyses for the Knowledge and Understanding domain. This finding may not be unusual, as an individual can possess knowledge regarding the benefits of adopting or altering certain behaviours yet decide not to act upon that knowledge [29]. Similarly, the fact that a child does not adhere to PAG or SBG may not be indicative of a lack of understanding of the concepts and accompanying principles that underlie a healthy active lifestyle (i.e., guidelines, health benefits, terminology). Other factors (such as motivation, enjoyment, parental support, and capability) may play a more significant role in the likelihood of children meeting PAG and SBG.

Only 20% (*n* = 577/2956) of participants in this study met the Canadian PAG. The prevalence of children meeting PAG from this cross-sectional study is higher than that found by the 2012–2014 wave of the Canadian Physical Activity Levels Among Youth (CANPLAY) study. The CANPLAY study, which also used pedometers to measure activity, found that only 7.8% of children met Canadian PAG [30]. This prevalence difference may be due to methodological factors, as our study did not use random sampling methods. Moreover, previous research has demonstrated that meeting PAG peaks in 9- to 10-year-olds [31] and our study’s sample included children aged 8–12 years. Therefore, our sample’s age range, which is more condensed than the CANPLAY study’s 5- to 19-year-old age range, may have led to a higher prevalence of children meeting PAG. Despite the difference in prevalence, the low percentage of Canadian children meeting PAG continues to be concerning, with the majority of children not obtaining health benefits associated with regular physical activity [6].

Regarding SBG, more than half of the study’s participants self-reported meeting SBG (57%, *n* = 1633). This study’s prevalence of meeting SBG is markedly higher than previous waves of nationally representative Canadian data (Canadian Health Measures Survey [CHMS] 2007–2009; 2009–2011; 2012–2013), which found through pooling the three survey periods that only 5.4% of 6- to 17-year-olds either self-reported or proxy-reported (parent-report via interview) meeting the guidelines [32]. However, the age range in the CHMS (6- to 17-year-olds) is much wider than the 8–12 age range in our study. In addition, research in Canada [33] and in the United States [21] indicates that screen time increases as children and adolescents become older. Thus, the greater prevalence of not meeting SBG in the older age groups from the CHMS may have reduced the overall average adhering to the guidelines, ultimately contributing to the disparity in comparison to our findings.

Though not a primary outcome of interest, significant gender differences were revealed after separately analyzing the associations between guideline adherence (i.e., PAG and SBG) and PL. Compared to girls, boys achieved significantly higher PL scores in all domains, with the exception of the Knowledge and Understanding domain, where no difference was observed. Furthermore, more boys met PAG, and the girls in this study had decreased odds of meeting PAG compared to boys. This is consistent with other national research in Canada, which found that Canadian boys took more daily steps on average than girls [34]. Conversely, more girls met SBG than boys, and girls were also at increased odds of meeting SBG. Previous research in Canada has also shown that boys engage in more screen time than girls [33], which is similar to the findings observed in our sample. Future research should expand on the potential gender differences between aspects of PL and adherence to PAG and SBG.

This study has strengths and limitations that warrant discussion. A marked strength is the large and geographically diverse sample of Canadian children participating in this study. An additional strength of this study was that the application of the CAPL was performed year-round to evaluate the impact of seasonality. Furthermore, the CAPL utilizes objective measures – administered by trained research staff – to measure the elements that comprise the Physical Competence and Daily Behaviour components of PL. In terms of limitations, Canadian children from the Northern territories (Yukon, Northwest Territories, Nunavut), Saskatchewan, New Brunswick, and Newfoundland & Labrador were not captured in this study. Consequently, this limits the applicability of the findings relative to Canadian children. Moreover, the data obtained from this study were from a cross-sectional design using convenience sampling methods, limiting the conclusions regarding the directionality of the associations. Pedometry was used to capture PA data instead of accelerometry, due to the surveillance study design, and this limited us from gathering any information on detailed movement counts (i.e., sedentary time, light PA, moderate to vigorous PA, etc.). Additionally, socio-economic status was not a measure of this study and could have influenced the findings.

## Conclusions

The primary outcome of this study was that children were at increased odds of meeting PAG and SBG if they achieved the minimum recommended level of PL domain scores, namely for Physical Competence and Motivation and Confidence. The implications of this research suggest that there are associations between children’s PL and the degree to which they adhere to PAG and SBG. Future research should account for limitations presented in this study and incorporate other factors, such as sociocultural, psychological and/or physical correlates, in prospective, longitudinal, and intervention studies.

## Abbreviations

CAMSA: Canadian Agility and Movement Skill Assessment
CAPL: Canadian Assessment of Physical Literacy
CHMS: Canadian Health Measures Survey
CI: Confidence interval
FMS: Fundamental movement skills
PAG: Physical activity guidelines
PL: Physical literacy
RBC Learn to Play - CAPL: Royal Bank of Canada Learn to Play – Canadian Assessment of Physical Literacy
SBG: Sedentary behaviour guidelines
